# Cyclic di-AMP as endogenous adjuvant enhanced BCG-induced trained immunity and protection against *Mycobacterium tuberculosis* in mice

**DOI:** 10.3389/fimmu.2022.943667

**Published:** 2022-08-23

**Authors:** Huanhuan Ning, Jian Kang, Yanzhi Lu, Xuan Liang, Jie Zhou, Rui Ren, Shan Zhou, Yong Zhao, Yanling Xie, Lu Bai, Linna Zhang, Yali Kang, Xiaojing Gao, Mingze Xu, Yanling Ma, Fanglin Zhang, Yinlan Bai

**Affiliations:** ^1^ Department of Microbiology and Pathogen Biology, School of Preclinical Medicine, Air Force Medical University, Xi’an, China; ^2^ College of Life Sciences, Northwest University, Xi’an, China; ^3^ Department of Endocrinology, Xijing Hospital, Air Force Medical University, Xi’an, China; ^4^ Department of Clinical Laboratory, Xijing Hospital, Air Force Medical University, Xi’an, China; ^5^ Laboratory Animal Center, Air Force Medical University, Xi’an, China; ^6^ School of Life Sciences, Yan’an University, Yan’an, China; ^7^ Department of Physiology, Basic Medical School, Ningxia Medical University, Yinchuan, China

**Keywords:** Bacillus Calmette-Guérin, cyclic di-AMP, trained immunity, adjuvant, *Mycobacterium tuberculosis*

## Abstract

Bacillus Calmette-Guérin (BCG) is a licensed prophylactic vaccine against tuberculosis (TB). Current TB vaccine efforts focus on improving BCG effects through recombination or genetic attenuation and/or boost with different vaccines. Recent years, it was revealed that BCG could elicit non-specific heterogeneous protection against other pathogens such as viruses through a process termed trained immunity. Previously, we constructed a recombinant BCG (rBCG-DisA) with elevated c-di-AMP as endogenous adjuvant by overexpressing di-adenylate cyclase of *Mycobacterium tuberculosis* DisA, and found that rBCG-DisA induced enhanced immune responses by subcutaneous route in mice after *M. tuberculosis* infection. In this study, splenocytes from rBCG-DisA immunized mice by intravenous route (i.v) elicited greater proinflammatory cytokine responses to homologous and heterologous re-stimulations than BCG. After *M. tuberculosis* infection, rBCG-DisA immunized mice showed hallmark responses of trained immunity including potent proinflammatory cytokine responses, enhanced epigenetic changes, altered lncRNA expressions and metabolic rewiring in bone marrow cells and other tissues. Moreover, rBCG-DisA immunization induced higher levels of antibodies and T cells responses in the lung and spleen of mice after *M. tuberculosis* infection. It was found that rBCG-DisA resided longer than BCG in the lung of *M. tuberculosis* infected mice implying prolonged duration of vaccine efficacy. Then, we found that rBCG-DisA boosting could prolong survival of BCG-primed mice over 90 weeks against *M. tuberculosis* infection. Our findings provided *in vivo* experimental evidence that rBCG-DisA with c-di-AMP as endogenous adjuvant induced enhanced trained immunity and adaptive immunity. What’s more, rBCG-DisA showed promising potential in prime-boost strategy against *M. tuberculosis* infection in adults.

## Introduction

Bacillus Calmette-Guérin (BCG) is a live attenuated vaccine for the prevention of tuberculosis (TB), which is caused by *Mycobacterium tuberculosis* infection. Usually, BCG is inoculated to newborns after birth, of which protection efficiency against meningitis and miliary TB is over 70% (1). Whereas its protection efficacy varies from 0% to 80% against pulmonary TB in adults (2). Vaccinology to improve the efficacy of canonical BCG shows good application prospects, which is necessary to control TB epidemic.

BCG was obtained from virulent *Mycobacterium bovis*, which could elicit heterologous protective immune response against *M. tuberculosis* infection. Additionally, BCG has been found to elicit non-specific protection against non-mycobacterial bacteria infections (3), viral infections (4), tumors (5), and autoimmune diseases (3). This phenomenon of BCG-induced heterologous protection has been referred to as trained immunity (6). The hallmarks of trained immunity include elevated levels of proinflammatory cytokine responses, myeloid cells and macrophage reprogramming towards inflammatory, activated phenotypes, epigenetic modifications, and metabolomic changes towards glycolysis (7–9). BCG vaccination shows long-term effects on innate immunity, which lasts for at least one year and then wanes in mice model (10). It is speculated that increasing morbidity of TB adults in BCG vaccinated population may be related to the waning efficiency of BCG (11, 12). Furthermore, BCG vaccination could promote protective monocyte responses against severe acute respiratory syndrome coronavirus 2 (SARS-CoV-2) (13, 14), though its protective effect on SARS-CoV-2 remains controversial (15, 16). Therefore, it is reasonable to increase the protection of BCG by exploring new BCG strains or booster vaccines to enhance BCG-induced trained immunity.

Cyclic di-AMP (c-di-AMP) is a ubiquitous bacterial second messenger (17, 18), which regulates various bacterial physiological processes including bacterial virulence. What’s more, there is growing evidence that c-di-AMP as a pathogen associated molecular pattern (PAMP) is engaged in host innate immune response including type I IFN response, autophagy, as well as inflammasome activation (19–21). Our previous work initially confirmed that Rv3586 is the only diadenylate cyclase (DacA, or DisA) (22), and Rv2837c is a cyclic nucleotide phosphodiesterase (CnpB) as a c-di-AMP hydrolyzing enzyme in *M. tuberculosis* (23). Either overexpression of DisA or deletion of CnpB could promote the accumulation of c-di-AMP in *M. tuberculosis* (23–25), BCG (24, 26, 27), as well as *M. smegmatis* (28–30). Further, we constructed a c-di-AMP elevated BCG with *disA* overexpressing (rBCG-DisA) and found that it induced stronger immune responses compared with BCG after *M. tuberculosis* infection in mice, with elevated cytokine responses of IL-6, TNF-α and enrichment of tri-methylation of lysine 4 on histone H3 (H3K4me3), which showed an enhanced trained immunity induced by rBCG-DisA (27).

However, we found that rBCG-DisA immunization by subcutaneous (s.c) route did not provide extra protection against *M. tuberculosis* in the mice model of i.v infection (27). While a similar rBCG-DisA with MT3692 (*M. tuberculosis* CDC1551 DisA, 100% similarity with Rv3586) overexpression has been shown to provide enhanced protection against aerosol *M. tuberculosis* infection compared to BCG in the guinea pig model (31). Moreover, rBCG-DisA induced improved antitumor efficacy by intravesical instillation in a rat model of bladder cancer, which was identified as an improved trained immunity compared with BCG in primary human and murine macrophages *in vitro* (32). It has been proved that BCG vaccination by i.v route is superior to induce trained immunity than that of s.c route (33). In addition, administration of BCG by i.v route conferred protection against *M. tuberculosis* challenge in non-human primate (34). In this study, we further explored immune responses induced by i.v rBCG-DisA especially trained immunity *in vivo*, and evaluated its protection efficiency and as a boosting vaccine for BCG-primed mice against *M. tuberculosis* intranasal (i.n) infection in mice model.

## Materials and methods

### Mice, bacterial strains, and cell lines

Six- to eight-week C57BL/6J male mice were purchased from Animal Center of Air Force Medical University. Recombinant BCG overexpressing DisA (termed as rBCG-DisA) was constructed in our previous work (27). *M. tuberculosis* H37Ra, BCG were grown in Middlebrook 7H9 broth (BD) supplemented with 0.5% (v/v) glycerol, 0.05% Tween 80 and 10% oleic acid albumin-dextrose-catalase (OADC) (BD) at 37°C. rBCG-DisA was grown in complete 7H9 medium with 25µg/mL kanamycin. All mycobacteria were plated on 7H10 agar (BD) supplemented with 10% OADC for colony forming units (CFUs) enumeration. *Staphylococcus aureus* was grown in LB medium at 37°C. Murine macrophage cell line RAW264.7 was used for *in vitro* experiments.

### Vaccination and infection of mice

Mice were anesthetized with an intraperitoneal injection of 50mg/kg pentobarbital sodium. Then, mice were vaccinated with 1×10^6^ CFU single bacterial suspension of BCG or rBCG-DisA in 200µL PBS at the base of the tail by i.v route, respectively (33). After 12 weeks, all vaccinated mice were challenged with 2×10^5^ CFU *M. tuberculosis* H37Ra in 50µL PBS by i.n route. Normal mice (Naïve) and *M. tuberculosis* H37Ra infected mice without vaccination (UN) were used as control. At 12 weeks post vaccination and 6 weeks post infection, mice were euthanatized for immunological assays, histopathological analysis and CFUs measurements.

### Antigen-specific antibody detection by ELISA

Sera from all mice were collected at indicated time points after vaccination or infection respectively. Enzyme linked immunosorbent assay (ELISA) microplates were coated with BCG or DisA antigen at 10µg/mL. Sera were diluted with PBS at indicated dilutions. Antigen-specific IgG level was measured by ELISA. The absorbance was detected at 450 nm by microplate reader (BioTek).

### Splenocytes preparation, stimulation, and detection of proliferation *in vitro*

Spleen single cell suspensions were prepared and 1×10^6^ splenocytes were seeded and stimulated by BCG proteins (25µg/mL) for 72h. Cell proliferation was determined by CFSE (Sigma) reagent according to previous study (27, 35). For cytokines measurement, 1×10^6^ cells were seeded in 96-well microplates stimulated with BCG proteins (25µg/mL), *M. tuberculosis* proteins (25µg/mL), *S. aureus* proteins (25µg/mL), *E. coli* O111:B4 lipopolysaccharide (LPS) (100ng/mL) (InvivoGen) for 72h, respectively. Cells were incubated at 37°C with 5% CO_2_ and supernatants were collected for cytokines assays.

### Determination of cytokines production by ELISA

Cytokines measurement in the cell supernatants was performed using commercial ELISA kits. Cytokines detection of IFN-γ, IL-2, IL-10, IL-17, TNF-α, IL-1β, IL-6 (from Thermo Fisher), CXCL9, CXCL10 and CXCL11 (CLOUD-CLONE CORP, China) were performed according to the instructions. Cell supernatants and diluted standards were added 100µL/well, then incubated at room temperature for 2h. Finally, ELISA plates were monitored at 450 nm using a microplate reader (BioTek).

### Flow cytometry

Splenocytes were washed with PBS containing 2.5% FBS. Cells were resuspended in 100μL Staining Buffer (BioLegend) containing TruStain Fc PLUS (anti-mouse CD16/32) (BioLegend) and incubated for 10min on ice. Cells were stained with fluorochrome-labeled antibodies of CD3 (17A2), CD4 (RM4-5), CD8 (53-6.7), CD19 (6D5), CD49 (DX5), CD11b (M1/70), Ly-6G (1A8) (BioLegend) for 30min at 4°C, then washed with PBS containing 2.5% FBS twice. Finally, cells were resuspended in 500µL Staining Buffer for flow cytometry analysis (BD FACSCanto). The frequency of specific cell subpopulation was analyzed using FlowJo V10 (Tree Star Inc.).

### Generation of BMDMs

After 4 weeks of vaccination, bone marrow from both femurs and tibiae was harvested in RPMI 1640. Cells were subsequently resuspended in RPMI 1640 supplemented with 10% FBS, 100U/mL penicillin, 100mg/mL streptomycin and 100ng/mL M-CSF (PeproTech). Cells were seeded in Petri dishes (100 mm). After 3 days of incubation at 37°C with 5% CO_2_, the medium was removed and replaced with fresh medium. Cells were cultured for another 2 days allowing to differentiate into macrophages. On day 5, cells were harvested and resuspended in complete RPMI 1640 medium for *in vitro* assay.

### Macrophage infection

RAW264.7 macrophages and BMDMs of 2×10^5^ were seeded in 24-well plates supplemented with complete RPMI 1640 without penicillin/streptomycin, and incubated at 37°C with 5% CO_2_ overnight. Cells were infected with *M. tuberculosis* attenuated strain H37Ra (MOI = 1), BCG and rBCG-DisA (MOI = 10). Cells were incubated for 4h at 37°C with 5% CO_2_. Subsequently, cells were washed with cold sterile PBS for 3 times, and then incubated in complete RPMI 1640 without penicillin/streptomycin. This time point was termed as 0 d post infection (dpi). Intracellular bacteria CFUs from lysed cells by 0.025% SDS were enumerated on plates at day 1, 3 and 5 post infection.

### Quantitative RT-PCR

Total RNA of mice tissues and bone marrow (BM) cells was extracted using the RNeasy kit (Omega Bio-Tek) following the manufacturer’s instruction, and quantified by microplate reader (BioTek). Next, elimination of genomic DNA and cDNA synthesis of 500 ng RNA were performed by HiScript Reverse Transcriptase Kit (Vazyme). Quantitative RT-PCR assay was performed using SYBR qPCR Master Mix (Vazyme). Fold changes of mRNA expression of indicated genes was calculated according to 2^-ΔΔCt^, gene of *gapdh* was used as expression normalization. Primers used in this study were synthesized by Tsingke Biological Technology and listed in **Table S1**.

### Histopathology and immunohistochemistry

For histopathology, upper lobes of left lungs and 1/3 of spleen tissue in infected mice was fixed in 10% buffered formalin. The tissue was dehydrated and embedded in paraffin wax. Sections (5µm) were cut and transferred onto glass slides. Hematoxylin/eosin (H&E) staining for pathohistological analysis was performed by the Department of Histopathology (Air Force Medical University, China), and results were quantified by Image J software.

The counts of CD4 and CD8 T cells and expression levels of H3 mono-, or tri-methylated at lysine 4 (H3K4me1, or H3K4me3) and H3K27 acetylation (H3K27ac) in the lung were determined by immunohistochemistry (IHC). Antibodies used in IHC assays were rabbit mAb anti-CD4 (D7D2Z) (CST), rabbit mAb CD8α (D4W2Z) (CST), rabbit mAb anti-Histone H3 (mono methyl K4) (Abcam), rabbit mAb anti-Histone H3 (acetyl K27) (Abcam), rabbit mAb anti-Histone H3 (tri methyl K4) (Abcam). Secondary antibody used was HRP-conjugated anti-rabbit antibody (Jackson Immunoresearch Laboratories). IHC assay was performed by Chengdu Lilai Biotechnology Company (Chengdu, China). IHC images were quantitatively analyzed with Image J software.

### Sera collection and extraction for metabolomics analysis

Whole blood samples from C57BL/6J mice were drawn at 6 weeks post *M. tuberculosis* infection. Sera were separated by centrifugation at 3 000 rpm for 5min after blood samples were incubated at 37°C for 30min. For LC-MS/MS analysis, 100µL serum was thoroughly mixed with 100µL pre-cooled water and 400µL cold methanol acetonitrile (v/v, 1:1) through vortex. Then, the mixture was processed with sonication for 1h on ice, and incubated at -20°C for 1h. The mixture was centrifuged at 4°C for 20min with 14 000g. The supernatants were harvested and dried under vacuum. 100 µL acetonitrile water solution (v/v, 1:1) was added to redissolve the samples, then centrifuged at 16 000g at 4°C for 20min. The supernatant was harvested for further analysis. Extraction of samples was performed by Bioprofile (Shanghai, China).

### Liquid chromatography tandem mass spectrometry (LC-MS/MS) based untargeted metabolomics analysis

For hydrophilic interaction liquid chromatography (HILIC) separation, samples were analyzed using a 2.1mm×100mm ACQUIY UPLC BEH Amide 1.7µm column (Waters, Ireland) by SHIMADZU-LC30 ultra-high performance liquid chromatography system (UHPLC, Shimadzu). Both electro-spray ionization (ESI) positive mode and negative mode were applied for MS data acquisition by QE Plus mass spectrometer (Thermo Fisher). The raw MS data were processed using MS-DIAL for peak alignment, retention time correction and peak area extraction. The metabolites were identified by accuracy mass (mass tolerance <0.01Da) and MS/MS data (mass tolerance <0.02Da) which were matched with Human Metabolome Database (HMDB) and Massbank databases. For the extracted data, ion peaks with the missing values >50% were deleted from the group. The total peak areas of positive and negative ion data were normalized, positive and negative ion peaks were integrated, and R software was used for pattern recognition. After the data were pretreated by Unit variance Scaling, subsequent data analysis was performed. LC-MS/MS based untargeted metabolomics analysis and data analysis were performed by Bioprofile (Shanghai, China).

### CFU enumeration and genotypic identification of bacilli by PCR

Lungs and spleens of mice were aseptically removed and homogenized through a 40µm cell strainer in 4mL RPIM 1640 medium. Then the tissue homogenates were serially diluted and plated on 10% OADC supplemented 7H10 agar plates containing polymyxin B sulfate (80µg/mL) and azlocillin sodium (5µg/mL) to avoid other bacterial contamination. Bacterial colonies were counted following 3-4 weeks of incubation at 37°C. The number of single colony of bacilli was recorded, and CFUs data are represented as Log_10_CFU. In order to distinguish *M. tuberculosis* H37Ra and BCG/rBCG-DisA, colonies on the plate were amplified by PCR with *ag85b* (present in both BCG and H37Ra) and *cfp10-esat-6* operon (only present in H37Ra) fragments (**Table S1**).

### Strategy of prime-boost and *M. tuberculosis* challenge

Female BALB/c mice were vaccinated with 1×10^7^ CFU of BCG by s.c route. At 58-week after vaccination, mice received a second boosting vaccination with the same dose and route of rBCG-DisA. Six weeks post boost, mice were challenged with 1×10^5^ CFU of *M. tuberculosis* H37Ra by i.n route. After infection, mice body weights were monitored weekly and survivals were recorded.

### Statistical analysis

Results were represented as mean values ± SEM. Statistical analysis of data performed by Graphpad Prism 9.0 Software (Graphpad Software, USA). Statistical significances were determined by two-tailed Student’s *t* test or for multiple comparisons by one-way ANOVA. For analysis of survival curves, we used log-rank test. The results were significant when *P < 0.05, **P < 0.01, ***P < 0.001, ****P < 0.0001 as given in the figure legends unless otherwise specified.

## Results

### rBCG-DisA elicited greater proinflammatory cytokine responses to homologous and heterologous re-stimulations than BCG

**Figure 1:**
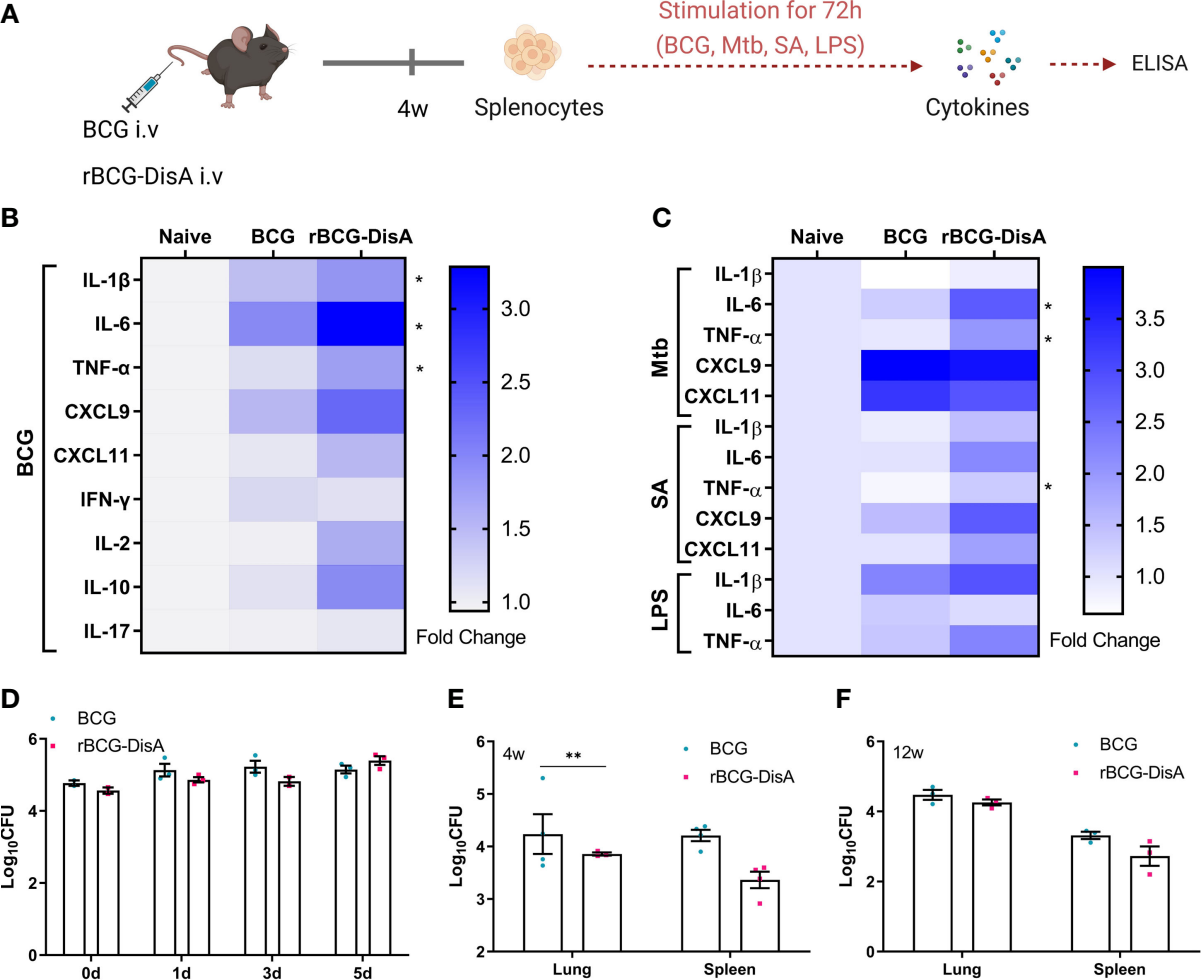
Inflammatory cytokine levels of rBCG-DisA i.v vaccinated mice and intracellular survival of rBCG-DisA in macrophages and mice. **(A)** Schematic diagram of homologous or heterologous stimulation of splenocytes *in vitro* (created with BioRender.com). After 4 weeks of BCG and rBCG-DisA i.v vaccination, splenocytes were isolated and re-stimulated with BCG protein extracts (BCG) (25μg/mL), *M. tuberculosis* (Mtb) (25μg/mL), *S. aureus* (SA) (25μg/mL), and *E. coli* O111:B4 LPS (LPS) (100ng/mL) for 72h *in vitro*. Normal mice (Naïve) were used as control. Supernatants were collected to measure cytokine secretion level by ELISA. **(B, C)** Cytokines and chemokines production of IL-1β, IL-6, TNF-α, CXCL9, CXCL11, IFN-γ, IL-2, IL-10 and IL-17 in supernatants were determined after re-stimulations by ELISA. The results are expressed as relative folds of each cytokine concentration (ng/mL) compared to the Naïve group (*n*=3). **(D)** Intracellular survival of BCG and rBCG-DisA in RAW264.7 macrophages at 0, 1, 3, and 5 days post infection (MOI=10). **(E, F)** The number of bacilli in the lung and spleen of mice was measured after 4 **(E)** and 12 **(F)** weeks of i.v immunization, respectively (*n*=3). (Panels **B, C**, ^*^ *P* < 0.05 stands for rBCG-DisA v.s BCG group. Panel **E**, ^**^ *P* < 0.01.).

Following exposure to infectious agents or vaccines, trained immunity can mount a faster and greater response against secondary challenge with homologous or even heterologous pathogens (8). Proinflammatory cytokines and chemokines have been reported as primary indicators of trained immunity, mainly including TNF-α, IL-1β, IL-6, CXCL9, CXCL10 and CXCL11 (36, 37). Both *in vitro* and *in vivo* experiments have shown that BCG immunization could induce increased extensive cytokine responses of IL-1β, TNF-α and IL-6 after training with BCG (33, 36, 38). It has been demonstrated that rBCG-DisA could elicit greater proinflammatory cytokines including IFN-β, IL-1β, IL-6 and TNF-α than that of BCG in primary human and murine macrophages (31, 32). In this study, splenocytes of immunized mice were re-stimulated with BCG, *M. tuberculosis*, *S. aureus* and *E. coli* LPS respectively (**Figure 1A**). BCG immunization by i.v route induced more IL-1β, IL-6, TNF-α, CXCL9 and CXCL11 releases to homologous stimulus re-stimulation in splenocytes of mice (**Figure 1B**). Our previous study found that rBCG-DisA immunization by s.c route only induced elevated IL-6 production in splenocytes of mice (27). Here, rBCG-DisA immunization induced elevated proinflammatory cytokines and chemokines of IL-1β, IL-6, TNF-α, CXCL9 and CXCL11 releases than that of BCG in splenocytes of mice responding to homologous antigens, and CXCL10 was under the limit of detection (**Figure 1B**).

Besides, BCG immunization increased the heterologous IFN-γ production and induced long lasting heterologous IL-17 responses in mice (39). Previously, we showed that splenocytes of rBCG-DisA s.c immunized mice produced Th1/Th2 cytokines of IFN-γ, IL-2 and IL-10 as that of BCG to homologous proteins (27). In this study, BCG immunization by i.v route induced slightly elevated IFN-γ and IL-10 production, while rBCG-DisA i.v immunization induced elevated IL-2 and IL-10 production to homologous proteins in splenocytes of mice (**Figure 1B**). It was found that BCG and rBCG-DisA did not induce IL-17 production to homologous proteins in splenocytes of mice after 4 weeks of immunization by i.v route (**Figure 1B**). rBCG-DisA immunization induced comparable levels of IFN-γ and IL-17 secretion after re-stimulated by homologous stimulus as that of BCG, but higher levels of IL-2 and IL-10 than that of BCG (**Figure 1B**).

It was proved that BCG trained innate immune cells showed specific responses according to different stimuli (37). Then splenocytes of immunized mice were re-stimulated with *M. tuberculosis*, *S. aureus*, and *E.coli* LPS respectively (**Figure 1A**). BCG immunization induced an enhanced IL-1β release to LPS, but not to *M. tuberculosis* and *S. aureus* (**Figure 1C**). rBCG-DisA immunization caused elevated releases of TNF-α to three bacterial components than that of BCG immunized mice, and IL-6 to *M. tuberculosis* and *S. aureus* (**Figure 1C**). Splenocytes from BCG and rBCG-DisA immunized mice produced significant chemokines of CXCL9 and CXCL11 responded to *M. tuberculosis* re-stimulation (**Figure 1C**). These data suggested that rBCG-DisA with c-di-AMP as endogenous adjuvant elicited enhanced proinflammatory cytokine responses in mice to homologous and heterologous stimuli.

Further, intracellular survival of BCG and rBCG-DisA in RAW264.7 macrophages were counted on plates. rBCG-DisA showed a slight decline on survival within 3 days, and increased at 5 days after infection, but no differences were found between two strains (**Figure 1D**). It was reported that a similar rBCG-DisA increased the levels of phagocytosis and autophagic processing within macrophages than BCG (32). After 4 weeks of immunization by i.v route, rBCG-DisA loads decreased in organs, especially in the lung of mice, but showed no difference compared with that of BCG in the lung and a slight decrease in the spleen after 12 weeks (**Figures 1E, F**). A latest study demonstrated that the similar rBCG-DisA based on another strain of Tice BCG proliferated to a lower degree in the lung of mice through aerosol inhalation (32). These results implied that enhanced cytokine responses of rBCG-DisA were not caused by different persistence of bacteria in host.

### rBCG-DisA immunization triggered similar cytokine responses in the spleen of mice with BCG did after *M. tuberculosis* challenge

**Figure 2:**
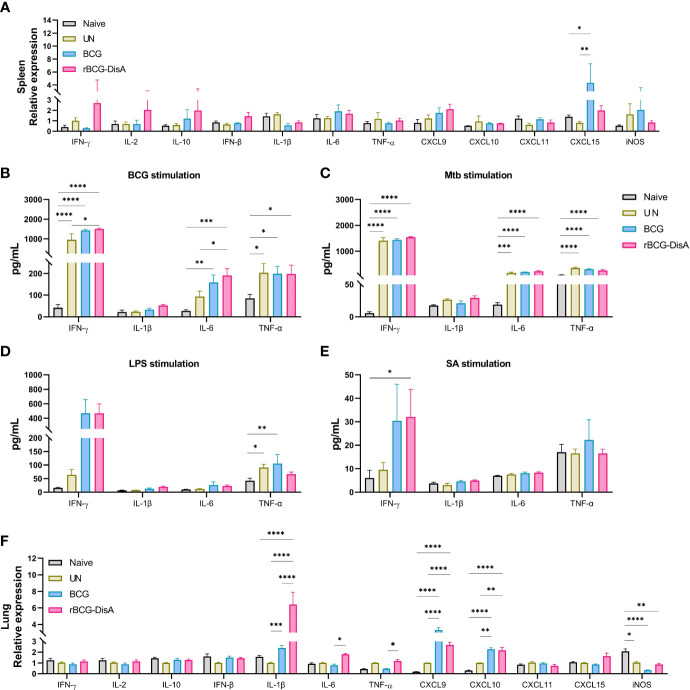
Inflammatory cytokines expression in the spleen and lung of vaccinated mice after *M. tuberculosis* infection. Mice were vaccinated by i.v BCG and rBCG-DisA for 12 weeks. Then, mice were infected with *M. tuberculosis* by i.n route, normal mice (Naïve) and *M. tuberculosis* infected mice without immunization (UN) were used as control. **(A)** At 6 weeks post infection, transcription level of indicated genes in the spleen of mice was assayed by quantitative RT-PCR (*n*=3). **(B–E)** At 6 weeks post infection, splenocytes were isolated and re-stimulated with BCG protein extracts (BCG) (25μg/mL) **(B)**, *M. tuberculosis* (Mtb) (25μg/mL) **(C)**, *E. coli* O111:B4 LPS (LPS) (100ng/mL) **(D)**, and *S. aureus* (SA) (25μg/mL) **(E)** for 72h *in vitro*. Cytokines production of IFN-γ, IL-1β, IL-6 and TNF-α in supernatants were determined by ELISA (*n*=4). **(F)** At 6 weeks post infection, transcription level of indicated genes in the lung of mice was assayed by quantitative RT-PCR (*n*=3). (^*^ *P* < 0.05, ^**^ *P* < 0.01, ^***^ *P* < 0.001,^****^ *P* < 0.0001.).

After 12 weeks of i.v immunization, mice were re-stimulated with *M. tuberculosis* H37Ra infection by i.n route (**Figure S1A**). At 6 weeks post *M. tuberculosis* challenge, spleen tissues were separated for detecting the transcription levels of cytokines and chemokines. As showed in **Figure 2A**, rBCG-DisA immunization induced similar transcription levels of almost all of proinflammatory cytokines and chemokines than un-immunized and BCG in the spleen after *M. tuberculosis* infection. Significant CXCL15 (IL-8) was reported before (36, 37, 40), which was not obvious in splenocytes of rBCG-DisA group. rBCG-DisA immunized mice showed an increasing trend on the transcription levels of IFN-γ, IL-2 and IL-10 in the spleen after *M. tuberculosis* infection, though no significant differences compared with that of UN and BCG immunized mice (**Figure 2A**). We speculated that splenocytes may not fully stimulated by i.n *M. tuberculosis in vivo* compared with proteins doses used *in vitro*. Further, we stimulated splenocytes from *M. tuberculosis* infected mice with homologous and heterologous stimuli. We found that rBCG-DisA immunization induced higher IL-6, IFN-γ and IL-10 secretions than BCG to homologous but not heterologous stimuli after *M. tuberculosis* infection (**Figures 2B–E**; **S1B–E**). We also showed that splenocytes from rBCG-DisA immunized mice and *M. tuberculosis* infected mice did not induce IL-1β to LPS stimulation, as UN and BCG groups did (**Figure 2D**).

### rBCG-DisA elicited greater inflammatory cytokines in the lung of mice after *M. tuberculosis* intranasal challenge

In this study, mice were immunized and then re-stimulated with *M. tuberculosis* infection by i.n route (**Figure S1A**). Lung tissues were also separated for detecting the transcription levels of cytokines and chemokines. rBCG-DisA i.v immunization significantly up-regulated proinflammatory cytokines of IL-1β, IL-6, and TNF-α in the lung than BCG i.v immunization after 6 weeks of *M. tuberculosis* challenge (**Figure 2F**). For chemokines, BCG and rBCG-DisA immunization promoted a similar increasing of CXCL9 and CXCL10, and rBCG-DisA immunization induced a slight increasing trend of CXCL15 expression in the lung after *M. tuberculosis* infection (**Figure 2F**). However, the results showed that the transcription levels of IFN-γ, IL-2 and IL-10 in the lung were no changes after *M. tuberculosis* infection in the lung of all groups (**Figure 2F**). These data suggested that rBCG-DisA immunization induced higher level of trained immunity in the lung, and were in line with such recombinant BCG that could trigger proinflammatory cytokine responses *in vitro* of BMDMs and macrophage cell line (31, 32).

### rBCG-DisA enhanced epigenetic changes than BCG in mice after *M. tuberculosis* challenge

**Figure 3:**
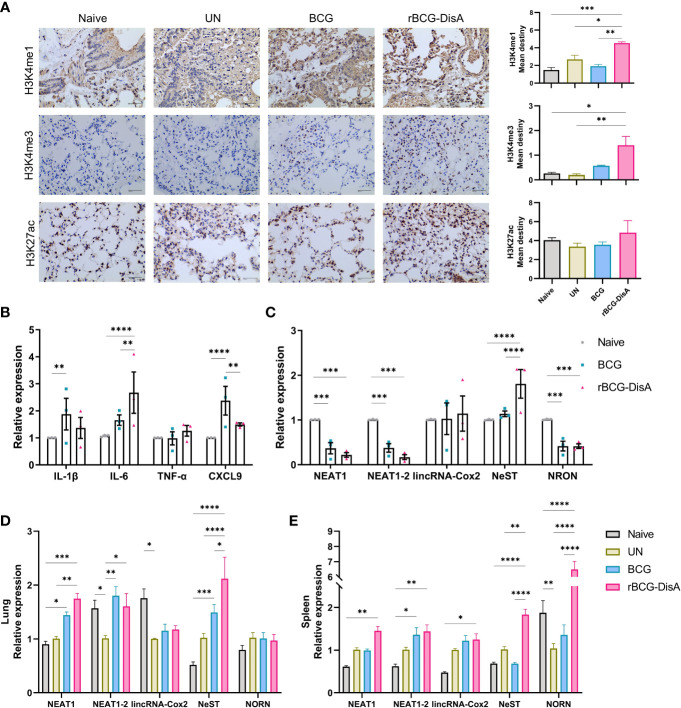
Expression of lncRNA and histone modifications in BM cells, lung and spleen of mice. Mice were vaccinated by i.v BCG and rBCG-DisA for 8 weeks (for BM cells isolation) and 12 weeks (for infection). Then, mice were infected with *M. tuberculosis* by i.n route, normal mice (Naïve) and *M. tuberculosis* infected mice without immunization (UN) were used as control. **(A)** At 6 weeks post infection, immunohistochemical analysis of H3K4me1, H3K4me3, and H3K27ac expression in the lung tissue (left). Quantitative analysis of H3K4me1, H3K4me3, and H3K27ac expression by Image J (right) (*n*=3). **(B, C)** At 8 weeks after BCG and rBCG-DisA i.v immunization, the expression of indicated cytokines **(B)** and non-coding RNA **(C)** in BM cells was detected by quantitative RT-PCR (*n*=3). **(D, E)** At 6 weeks post infection, immune responses were detected. Levels of indicated noncoding RNA in the lung **(D)** and spleen **(E)** were assayed by quantitative RT-PCR (*n*=3). (^*^ *P* < 0.05, ^**^ *P* < 0.01, ^***^ *P* < 0.001, ^****^ *P* < 0.0001.).

More data showed BCG is an inducer of trained immunity through increased transcription of proinflammatory cytokine and chemokine genes by epigenetic modifications (33, 41). It has been proved that epigenetic signatures of H3K4me3 and H3K27ac would decrease gradually with BCG inoculation time, and H3K4me1 persisted for long time to form immune memory (42, 43). Previously, we had identified that rBCG-DisA immunization by s.c route induced an enhanced H3K4me3 in the lung of mice after 4 weeks of *M. tuberculosis* infection (27). In this study, mice were immunized by i.v route for 12 weeks and challenged with *M. tuberculosis* (**Figure S1A**). Above, we had already established that rBCG-DisA acted as a more potent inducer of TNF-α and IL-6 production than BCG after re-stimulations *in vitro* (**Figures 1B, C**) and in the lung of mice (**Figure 2F**). Further, we detected epigenetic signatures of BCG-induced trained immunity including H3K4me3, H3K4me1 and H3K27ac by IHC in the lung of *M. tuberculosis* infected mice. It was shown that rBCG-DisA immunization by i.v route could obviously elicit enhanced epigenetic changes of H3K4me1 and H3K4me3, and a mild increasing in H3K27ac (**Figure 3A**), which suggested that rBCG-DisA elicited extended epigenetic changes compared with BCG, especially H3K4me1 for long time of immune memory.

### rBCG-DisA caused lncRNA expression changes in BM cells as well as in the lung of mice after *M. tuberculosis* challenge as BCG

It has been reported that i.v BCG could access bone marrow to expand hematopoietic stem cells (HSCs) and promotes myelopoiesis, and educated HSCs to generate trained monocytes/macrophages (33). To further characterize the trained immunity-inducing potential of rBCG-DisA versus BCG, we investigated the cytokine and chemokine expressions of BM cells from mice after 8 weeks i.v immunization. It was found that enhanced IL-6 expression was induced in BM cells of rBCG-DisA immunized mice compared with Naïve and BCG (**Figure 3B**). Whereas, rBCG-DisA immunization did not trained BM cells to express more IL-1β and CXCL9 as BCG did (**Figure 3B**). There were no significant differences in expressions of other cytokines and chemokines including IFN-γ, IFN-β, CXCL10, CXCL11, CXCL15 and iNOS (**Figure S2**). These results suggested BCG and rBCG-DisA immunization elicited different trained immune responses in central immune organ in mice.

Long noncoding RNAs (lncRNAs) are increasingly appreciated as regulators of cell-specific gene expression, which may involve in regulation of trained immunity. It has been identified that a lncRNA, named as stream master lncRNA of the inflammatory chemokine locus (UMLILO), could be brought proximal to immune genes (CXCL1, CXCL2, CXCL3, and CXCL15) prior to their activation and involve in innate immune defense (44). Since UMLILO is absent in mice, we detected some candidates lncRNAs that mediates expressions of immune response genes. It was found that lncRNA cyclooxygenase-2 (cox-2) (lincRNA-Cox2) was significantly increased in patients and macrophages with *M. tuberculosis* H37Ra infection (45). Both BCG and rBCG-DisA just induced a slight increase of lincRNA-Cox2 expression in BM cells from immunized mice (**Figure 3C**). After *M. tuberculosis* challenge, lincRNA-Cox2 expression level in the lung decreased, while the expression level in the spleen remained unchanged (**Figures 3D, E**).

It was reported that lncRNA NEAT1 (nuclear paraspeckle assembly transcript 1) was highly expressed in PBMCs and granulomatous tissue from TB patients, as well as in *M. tuberculosis* infected macrophages, and declined gradually with treatment (46). LncRNA NEAT1 appears in two isoforms of NEAT1-1 (short isoform) and NEAT1-2 (long isoform). We found that both BCG and rBCG-DisA seemed to inhibit NEAT1 expression in BM cells of immunized mice (**Figure 3C**). NEAT1 expression levels were elevated in the lung of BCG and rBCG-DisA immunized mice after *M. tuberculosis* infection (**Figure 3D**). Noticeably, rBCG-DisA immunization induced significant NEAT1 expression in the lung and spleen compared with that of BCG (**Figures 3D, E**). And there were no differences of NEAT1-2 expression levels between mice with BCG and rBCG-DisA immunizations (**Figures 3D, E**).

LncRNA NeST (Tmevpg1, abbreviates Nettoie Salmonella pas Theiler’s) is located in the nucleus, which is an enhancer lncRNA. In activated CD8^+^ T cells, NeST could recruit mixed lineage leukemia protein (MLL) and transactivate IFN-γ by binding to the adaptor protein WD repeat-containing protein 5 (WDR5) (47). It was observed that rBCG-DisA immunization elicited significant NeST transcription in BM cells of mice than that of BCG (**Figure 3C**). Similar increases were observed in the lung and spleen of rBCG-DisA i.v mice post *M. tuberculosis* infection (**Figures 3D, E**).

LncRNA NRON (noncoding repressor of nuclear factor of activates T cells ^[NFAT]^) is highly expressed in resting CD4^+^ T lymphocytes, which potently suppresses the latent HIV-1 transcription (48). Our data revealed that both BCG and rBCG-DisA i.v vaccination repressed the expression of NRON in BM cells (**Figure 3C**). After *M. tuberculosis* infection, rBCG-DisA increased the level of NRON than BCG in the spleen but not in the lung (**Figures 3D, E**).

### rBCG-DisA vaccination leads to an altered metabolic profile after *M. tuberculosis* challenge

**Figure 4:**
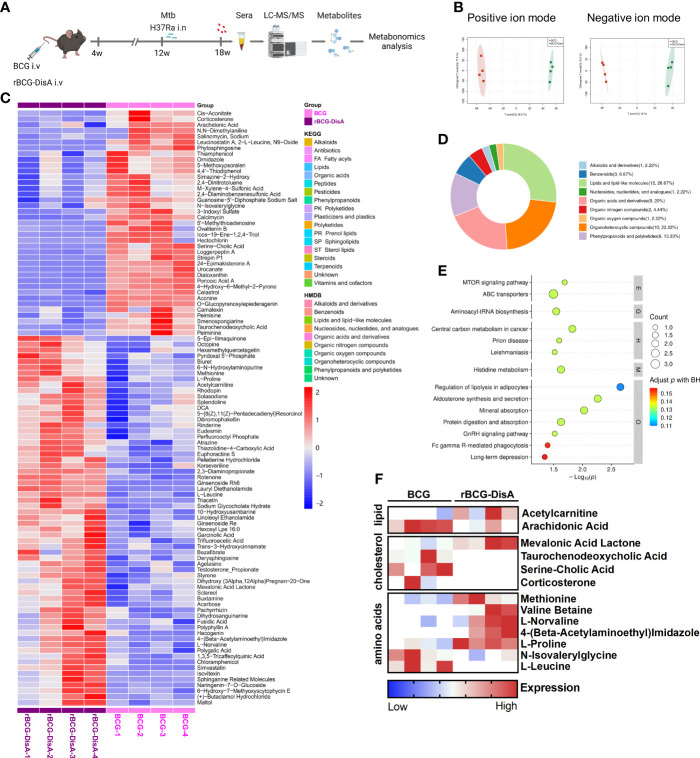
Metabonomics analysis of mice vaccinated with BCG or rBCG-DisA after *M. tuberculosis* infection. Mice were vaccinated by BCG and rBCG-DisA for 6 weeks. Then, mice were infected with *M. tuberculosis* by i.n route, normal mice (Naïve) and *M. tuberculosis* infected mice without immunization (UN) were used as control. At 8 weeks post infection, sera were harvested for metabonomics analysis (*n*=4). **(A)** A scheme diagram of detection on sera of mice vaccine with BCG or rBCG-DisA (created with BioRender.com). **(B)** OPLS-DA score plots of positive and negative ion modes of BCG and rBCG-DisA groups (*n*=4). **(C)** Heatmap of differential expressed metabolites of rBCG-DisA v.s BCG (*n*=4). **(D)** Circle diagram of metabolites classification of rBCG-DisA v.s BCG (*n*=4). **(E)** KEGG pathway enrichment of metabolites of rBCG-DisA v.s BCG (*n*=4). **(F)** Heatmap of differential expressed metabolites regarding lipid, cholesterol and amino acids metabolism of rBCG-DisA v.s BCG (*n*=4).

Metabolic rewiring is the major signature of BCG-induced trained immunity. We collected sera at 8 weeks post infection for untargeted metabolomics analysis based on LC-MS (**Figure 4A**). To reveal the changes of metabolites in BCG or rBCG-DisA immunized mice, supervised multivariate orthogonal partial least squares discriminate analysis (OPLS-DA) models were constructed to discriminate the differentiating variables. The results showed that rBCG-DisA group was obviously separated from the BCG group (**Figure 4B**), indicating that these two groups had different metabolic profiles in sera. Among these differentially expressed metabolites of rBCG-DisA v.s BCG, first proportion (26.67%) belonged to lipids or lipid-like molecules, and the subsequent proportions were organic acids or organic nitrogen molecules, respectively (**Figures 4C, D**). Through KEGG analysis, it was found that metabolites were mainly enriched in metabolic pathways such as lipolysis, aldosterone synthesis, protein digestion and absorption, etc. (**Figure 4E**). As shown in **Figure 4F**, the level of acetylcarnitine was up-regulated in rBCG-DisA immunized mice after *M. tuberculosis* infection, which helps long-chain fatty acyl-CoA to enter mitochondria, indicating that fatty acid oxidation and energy metabolism were promoted (49). Arachidonic acid, which inhibits fat mobilization, was also down-regulated in rBCG-DisA-immunized mice (**Figure 4F**). Thus, rBCG-DisA immunization promoted fat mobilization and lipolysis in mice.

### rBCG-DisA repressed specific IgG and CD4^+^ T cells by intravenous immunization

**Figure 5:**
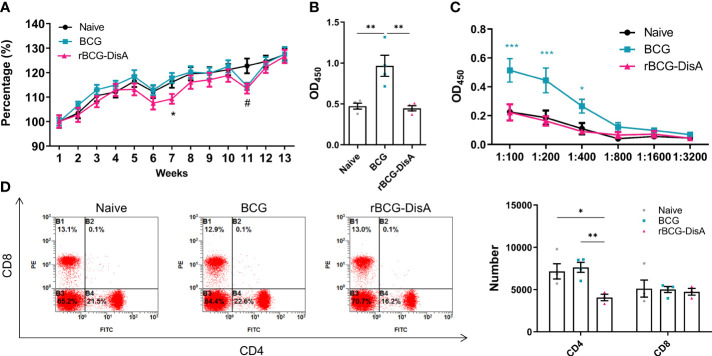
Adaptive immune response induced by rBCG-DisA vaccination. Male C57BL/6J mice were vaccinated with 1×10^6^ CFU BCG and rBCG-DisA by i.v route. At 4- and 12-week post vaccination, immune responses were determined. Normal mice (Naïve) were used as control. **(A)** Dynamic monitoring of mice body weights at each week after immunization. Data were presented as a percentage of body weight to initial body weight (*n*=3). **(B)** After 4 weeks of i.v immunization, BCG-specific IgG levels in mice sera (1:200) were assayed by ELISA (*n*=4). **(C)** After 12 weeks of i.v immunization, BCG-specific IgG titers in sera of mice at indicated dilutions were assayed by ELISA (*n*=4). **(D)** After 4 weeks of i.v immunization, CD4 and CD8 T cells in splenocytes were analyzed by flow cytometry of immunized mice (left). Numbers of CD4 and CD8 T cells in splenocytes were measured by flow cytometry (*n*=4) (right). (Panel **A**, ^*^ *P* < 0.05 stands for rBCG-DisA v.s BCG, ^#^ *P* < 0.05 stands for rBCG-DisA v.s Naïve. Panel **C**, ^*^ *P* < 0.05 and ^***^ *P* < 0.001 stand for rBCG-DisA v.s BCG/Naïve. Panel **D**, ^*^ *P* < 0.05, ^**^ *P* < 0.01.).

It is concluded that the heterologous protection conferred by BCG is likely the result of two mechanisms that synergize to induce protection: heterologous T cell immunity and trained immunity (3, 39). Previously, we found that rBCG-DisA s.c administration induces comparable humoral and cellular immune responses as BCG did in mice (27). We further analyzed the level of adaptive immune response in mice vaccinated with i.v BCG and rBCG-DisA strains. During the whole experiment, mice showed similar general behaviors such as spontaneous behavior, arousal behavior, grooming behavior, fecal traits among Naïve, BCG and rBCG-DisA groups. The body weights of BCG group mice increased steadily comparable to Naïve mice within 12 weeks after vaccination (**Figure 5A**). rBCG-DisA vaccinated mice showed lower weight gain than control and BCG mice (**Figure 5A**).

BCG administrated by i.v route induced higher mycobacteria-specific humoral immune response (**Figures 5B, C**; **S3A**). Whereas, rBCG-DisA i.v vaccination may repress the increasing of IgG than BCG 4-week and 12-week post vaccination (**Figures 5B, C**, **S3A**). BCG vaccination induced long-lasting effects of Th1/Th17 responses, and T-cell subpopulations did not show major shifts in CD4 and CD8 lymphocytes for at least one year in healthy volunteers (39). The proliferation of mice splenocytes were comparable in both two vaccinated groups compared to that of Naïve mice after re-stimulated by mycobacteria antigens by CFSE staining (**Figure S3B**). We found that rBCG-DisA immunization by s.c route increased the proportion of CD4^+^ but not CD8^+^ T cells (27). Whereas, rBCG-DisA vaccination by i.v route reduced the number and proportion of CD4^+^ T cells compared to both control and BCG groups (**Figure 5D**, **S3D**), and no significant changes on CD8^+^ T cells (**Figure 5D**, **S3C**).

### rBCG-DisA vaccination increased the CD4^+^ and CD8^+^ T cells after *M. tuberculosis* infection

**Figure 6:**
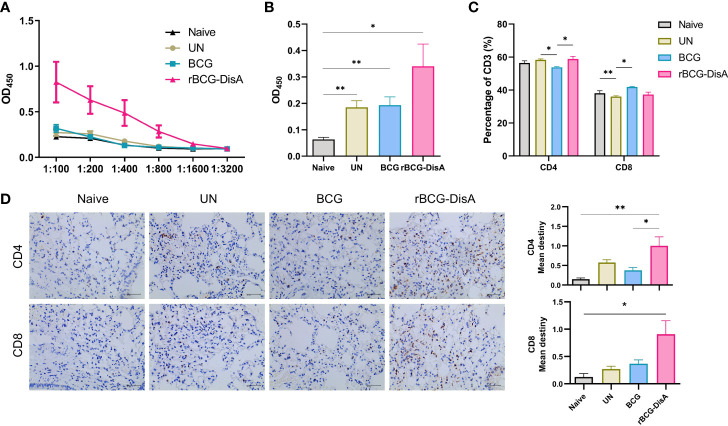
Analysis of specific antibodies and T cells in the spleen and lung of vaccinated mice after *M. tuberculosis* infection. Mice were vaccinated by i.v BCG and rBCG-DisA for 12 weeks. Then, mice were infected with *M. tuberculosis* by i.n route, normal mice (Naïve) and *M. tuberculosis* infected mice without immunization (UN) were used as control. **(A)** At 6 weeks post infection, BCG-specific IgG titers in sera were determined by ELISA at indicated dilutions (*n*=4). **(B)** At 6 weeks post infection, DisA-specific IgG levels in sera was determined by ELISA at dilution of 1:200 (*n*=4). **(C)** At 6 weeks post infection, proportions of CD4 and CD8 T cells in splenocytes were assayed by flow cytometry (*n*=4). **(D)** At 6 weeks post infection, immunohistochemical analysis of CD4 and CD8 T cells in the lung of mice (left). Quantitative analysis of CD4 and CD8 T cells by Image J (right) (*n*=3). (^*^ *P* < 0.05, ^**^ *P* < 0.01.).

After 12 weeks of i.v immunization, mice were infected with *M. tuberculosis* H37Ra by i.n route (**Figure S1A**). Six weeks post *M. tuberculosis* challenge, BCG i.v immunized mice exhibited low mycobacteria-specific IgG as Naïve and *M. tuberculosis* H37Ra i.n infection (UN) mice (**Figure 6A**). rBCG-DisA vaccination elicited stronger BCG- and DisA-specific IgG than that of BCG (**Figures 6A, B**). To our surprise, all infected mice showed similar percentages of T cell, B cell, NK cell, macrophage and neutrophil with Naïve mice (**Figure S4**). Further analysis of T cell subsets found that *M. tuberculosis* H37Ra i.n infection (UN) had negligible effect on proportions of CD4^+^ T cells, but caused a significant decline of CD8^+^ T cells in splenocytes (**Figure 6C**). BCG i.v immunized mice showed a decline of CD4^+^ T cells compared with UN group, but rBCG-DisA didn’t (**Figure 6C**). Both BCG and rBCG-DisA immunization resisted the reduce of CD8^+^ T cells caused by *M. tuberculosis* infection, and BCG showed a significant increase compared with UN group (**Figure 6C**). rBCG-DisA immunization kept the percentage of CD8^+^ T cells in splenocytes after *M. tuberculosis* infection closed to that of Naïve mice (**Figure 6C**).

Above results showed that rBCG-DisA i.v vaccinated mice exhibited greater innate immune inflammatory responses in the lung after *M. tuberculosis* infection (**Figure 2F**). Further, adaptive immune response in the lung was observed through the distribution of CD4^+^ and CD8^+^ T cells in the lung by IHC after *M. tuberculosis* infection by i.n route. As illustrated in **Figure 6D**, BCG i.v immunization caused similar T cells distribution after *M. tuberculosis* infection with UN group. More importantly, it was clearly showed that increased of CD4^+^ and CD8^+^ T cells in the lung of rBCG-DisA immunized mice after *M. tuberculosis* i.n infection (**Figure 6D**) implying significant T cell infiltration against infection.

### rBCG-DisA caused similar pathological changes as BCG after *M. tuberculosis* intranasal challenge

**Figure 7:**
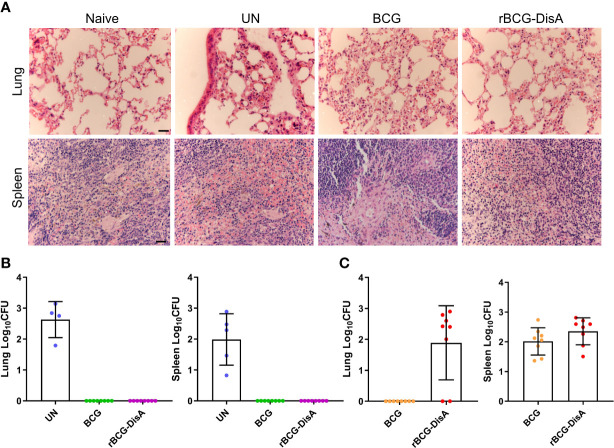
Histopathological changes and bacilli number of mice after *M. tuberculosis* infection. Mice were vaccinated by i.v BCG and rBCG-DisA for 12 weeks. Then, mice were infected with *M. tuberculosis* by i.n route, normal mice (Naïve) and *M. tuberculosis* infected mice without immunization (UN) were used as control. **(A)** At 6 weeks post infection, representative images of H&E-stained lung tissue sections at ×40 magnification (scale bar=100 μm). **(B, C)** At 6 weeks post infection, bacilli burdens in the lung and spleen were incubated at 37°C for 3-4 weeks (*n*=4~8). The colonies on the plates were determined according to the time of colony formation, morphology and genotype verification to determine whether the clone was *M. tuberculosis* [*ag85B* (+), *cfp10-esat-6* (+)] **(B)** or BCG/rBCG-DisA [*ag85B* (+), *cfp10-esat-6* (-)] **(C)**.

Since trained immunity confers broad immunological protection, it was worried that enhanced immune response of reprogrammed innate immune cells might result in the development or persistence of chronic metabolic, autoimmune or neuro-inflammatory disorders (50). rBCG-DisA induced stronger innate and adaptive immune responses, and did not cause excessive immunopathological damages by s.c inoculating in mice or intradermally route in guinea pig model (27, 31). Recently, the latest research demonstrates that a similar rBCG-DisA with different BCG strain was less pathogenic in immunocompromised SCID mice (32). As shown in **Figure 7A**, we did not observe the characteristics of tuberculosis such as tubercle and caseous necrosis in both lung and spleen tissues of infected mice. In the lung, *M. tuberculosis* infected mice exhibited obviously inflammatory cells infiltration (**Figure 7A**). In the lung of BCG and rBCG-DisA immunized mice, the alveolar structures were intact basically, accompanied with occasional thickening of alveolar mesenchyme, inflammatory cell infiltration, erythrocyte and histological fluid exudation (**Figure 7A**). Additionally, spleen of two immunized groups showed inflammatory cell infiltration as un-immunized mice after *M. tuberculosis* infection (**Figure 7A**). Overall, the inflammatory manifestations in the lung were comparable between rBCG-DisA and BCG vaccinated mice after *M. tuberculosis* challenge.

### rBCG-DisA provided similar protection against *M. tuberculosis* intranasal infection and persisted longer in the lung than BCG

After 12 weeks of immunization by i.v route, mice were infected with *M. tuberculosis* H37Ra infection by i.n route (**Figure S1A**). Six weeks post *M. tuberculosis* H37Ra i.n infection, the number of bacilli in the spleen and lung of mice was enumerated on plates. Considering that bacilli colonies could be *M. tuberculosis*, BCG or rBCG-DisA, we randomly selected numbers of bacterial clones for identification with specific primers (**Table S1**). The results showed that *M. tuberculosis* H37Ra were not identified under our detection method in the spleen and lung of BCG and rBCG-DisA immunized mice (**Figure 7B**). While, genotype identification of bacillus showed that the clones in two immunized groups were BCG or rBCG-DisA strains (**Figure 7C**). Further, we found BCG below the limit of detection in the lung after *M. tuberculosis* infection, but only two of eight mice showed non-sterilized of rBCG-DisA (**Figure 7C**). While the distribution of BCG and rBCG-DisA were similar in the spleen after H37Ra challenge (**Figure 7C**). These results suggested that rBCG-DisA immunization by i.v route could provide long-term protection than BCG after *M. tuberculosis* infection, which may be resulted from longtime survival of rBCG-DisA in the lung.

### rBCG-DisA boosting prolonged the lifespan of BCG-primed mice against *M. tuberculosis* intranasal infection

**Figure 8:**
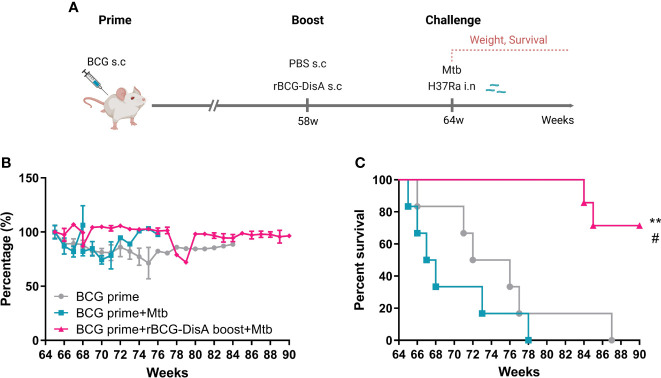
Body weight and survival in BCG prime following rBCG-DisA boost mice after *M. tuberculosis* infection. **(A)** BALB/c mice were primed by s.c BCG for 58-week following s.c rBCG-DisA boosting for 6-week. Subsequently, mice infected with *M. tuberculosis* by i.n route. Body weight and survival of mice were continuously monitored post *M. tuberculosis* infection. The schematic diagram of prime-boost strategy was created with BioRender.com. **(B)** Dynamic monitoring of mouse body weight at each week after *M. tuberculosis* infection. Results are presented as a percentage of body weight to initial body weight. **(C)** Survival of mice after *M. tuberculosis* infection (*n*=5~6). (Panel **C**, **P < 0.01 stands for rBCG-DisA boosting group v.s BCG primed group. ^#^ *P* < 0.05 stands for rBCG-DisA boosting group v.s and BCG primed+Mtb group.).

BCG-induced trained immunity gradually declines over time and vaccination protection declines with time, so a booster vaccine may be given in adolescence or adults when the effects of BCG may start to wane (11, 12). In this study, BALB/c mice were primed by s.c BCG for 58 weeks, then mice were boosted with rBCG-DisA at the same route (**Figure 8A**). After 6 weeks, mice were challenged by *M. tuberculosis* H37Ra i.n infection (**Figure 8A**). rBCG-DisA boosted mice showed a weight maintenance within 9 weeks infection, compared with weight loss in BCG-primed with or without *M. tuberculosis* infected mice (**Figure 8B**). We observed a significant longevity in rBCG-DisA boosted mice compared to only BCG primed mice after *M. tuberculosis* i.n challenge (**Figure 8B**). BCG primed mice all died within 14 weeks after *M. tuberculosis* infection, and all non-infected mice died within 87 weeks (**Figure 8C**). Surprisingly, until now 66% of rBCG-DisA boosted mice were alive over 90 weeks (**Figure 8C**). Thus, rBCG-DisA may be used as a candidate vaccine for BCG prime-boost regimens against *M. tuberculosis* infection in adults.

## Discussion

In the previous study, we found that rBCG-DisA by s.c route induced stronger immune responses compared with BCG in mice, which suggested an enhanced trained immunity was induced in mice (27). It has been proved that BCG accesses to BM and educate HSCs to generate epigenetically modified macrophages, and provide protective innate immunity against *M. tuberculosis* through trained immunity (33). In this study, we provided *in vivo* experimental evidence that rBCG-DisA with c-di-AMP as endogenous adjuvant could induce enhanced trained immunity and concomitant activated adaptive immune response, which showed a significant protection against *M. tuberculosis* infection in prime-boost strategy in adult mice.

Trained monocytes and macrophages display functional and epigenetic reprogramming, leading to increased production of cytokines and chemokines, which are viewed as primary indicators of trained immunity (37), and improved phagocytosis and killing capacity (51, 52). c-di-AMP has been identified to be a potent inducer of several proinflammatory cytokines and chemokines such as IL-1β, IL-6, and TNF-α in macrophages (27, 31, 32, 53). These proinflammatory cytokines are also considered as mediators of trained immunity (8, 9, 54, 55). It has been reported that rBCG-DisA could elicit greater proinflammatory cytokines than that of BCG in primary human and murine macrophages (27, 31, 32). Our data showed that BCG and rBCG-DisA elicited proinflammatory cytokines and chemokines transcriptions with different models in splenocytes of immunized mice, according to homologous or heterologous stimuli (**Figures 1B, C**). Remarkably, rBCG-DisA immunization induced enhanced proinflammatory cytokines, chemokines and Th1/Th2 cytokines transcriptions to re-stimulations *in vitro* than BCG in splenocytes of immunized mice (**Figure 1B**), and significant proinflammatory cytokines transcriptions in the lung to re-stimulation of *M. tuberculosis* i.n infection (**Figure 3F**). However, rBCG-DisA did not improve phagocytosis and killing capacity of macrophages than that of BCG as other inducer of trained immunity (**Figure 1D**) (51).

Generally, the induction of trained immunity is accompanied with the epigenetic modification at the promoters of cytokines such as IL-1β, IL-6, and TNF-α (56). Accumulations of H3K4me1 and H3K4me3 caused by rBCG-DisA may contribute to higher IL-6 than BCG in BM cells (**Figures 2F**, **3A**). However, the molecular mechanism of histones regulating the expression of inflammation-related genes have not yet been elucidated. H3K4me3 is directed to specific promoters in the genome by the presence of a class of lncRNAs, called immune gene-proximal lncRNAs (IPLs), which to be brought proximal to immune genes prior to their activation (44). UMLILO is the first reported IPL, which acts in *cis* to direct the WDR5MLL1 complex across the chemokine promoters (44). Surprisingly, an enhancer lncRNA of NeST, which binding to the adaptor protein WDR5 (47), was greater up-regulated in BM cells of rBCG-DisA i.v immunized mice than that of BCG, as well as in the lung and spleen after *M. tuberculosis* i.n infection (**Figures 3C–E**). IPLs are transcribed in a NFAT. Silencing of IPLs or abrogation of NFAT signaling results in loss of H3K4me3 accumulation at trained immune genes (44). We found that lncRNA NRON was repressed in BM cells of both BCG and rBCG-DisA i.v immunized mice, and showed no change in the lung but up-regulated in the spleen of rBCG-DisA i.v immunized mice to re-stimulation of *M. tuberculosis* infection (**Figures 3C–E**). Then we found that the expression of NEAT1 was down-regulated in BM cells of both BCG and rBCG-DisA immunized mice, but up-regulated significantly in the organs of rBCG-DisA immunized mice after *M. tuberculosis* challenge (**Figures 3C–E**). A growing body of research has revealed abnormally expressed lncRNAs in macrophages of TB patients (57). Thus, these lncRNAs that screened in this study may play roles in the regulation of the trained immunity, which should be investigated further.

Studies have demonstrated that immunological signals, metabolic rewiring of cell metabolism, and epigenetic reprogramming are integrated, representing the molecular substrates for induction of trained immunity (52). A recently research reported that another DisA overexpressing BCG (BCG Tice strain) elicited increased glycolytic metabolites, reduced kynurenine accumulation and itaconate production than wild type BCG in human or murine macrophages (32). However, these observations usually were generated *in vitro* model with different types of macrophages, which are known to arise from distinct cell lineages emerging at different stages in embryonic development. Thus, *in vivo* reality is of much greater nuance and complexity than can be accommodated by a macrophages model. In this study, we checked the metabolic profiles of mice immunized by BCG and rBCG-DisA after *M. tuberculosis* infection by metabolomic analysis, and found that increased fatty acid oxidation, fat mobilization, lipolysis and energy metabolism, as well as mTOR activation (**Figure 4**). It has been confirmed that induction of trained immunity accompanied by activated STING with enhanced mTOR-HIF-1α pathway activation and concomitant elevation in glucose transporter levels (58–60), which implied that rBCG-DisA promoted glycolysis in mice post *M. tuberculosis* infection. Besides, mTOR has been implicated in both the breakdown and synthesis of fatty acids, and rBCG-DisA immunized mice presented fatty acid oxidation trend. Thus, rBCG-DisA vaccination increased the catabolic signatures of fat mobilization and lipolysis than BCG in mice after *M. tuberculosis* infection. Cholesterol metabolism is a part of lipid metabolism. BCG-induced activation of TCA cycle promotes acetyl-CoA production, causes mevalonate accumulation, which promotes cholesterol synthesis (61). rBCG-DisA induced down-regulation of taurochenodeoxycholic acid, serine-cholic acid, and corticosterone, while the expression of mevalonic acid lactone inhibiting mevalonic acid synthesis was up-regulated (**Figures 4C, F**), suggesting that cholesterol synthesis in rBCG-DisA immunized mice was inhibited compared with BCG. Besides, rBCG-DisA induced up-regulation of essential amino acids and their metabolites such as methionine, valine betaine, L-norvaline valine, etc. Meanwhile, the expression of non-essential amino acid and its metabolites like L-proline, 4-(beta-acetylaminoethyl) imidazole (also called N-acetylhistamine) increased. The level of essential amino acids is positively correlated with activation of immune system induced by *M. tuberculosis* (62). And N-acetylhistamine may be related to anaphylaxis (63). Thus, shifts in metabolite levels may be a plausible mechanism behind the integration of immuno-metabolic and epigenetic programs for the enhanced trained immunity induced by rBCG-DisA immunization in mice.

Trained immunity is mediated by innate immune cells such as monocytes, macrophages, or NK cells (64), which produce heterologous lymphocyte activation, resulting in enhanced proinflammatory cytokines production, macrophage activity, T cell responses, and antibody titers (52). Activation of surface molecules such as pattern recognition receptor (PRR) and signaling pathways leading to the induction of Th1/Th2/Th17 response during trained immunity (39, 64). BCG could drive a robust mycobacteria specific antibody response in plasma and lungs, including IgG1, IgA, and IgM by i.v route, which could provide protection to an attenuated strain of *M. tuberculosis* (Δ*sigH* in the *M. tuberculosis* CDC1551) in macaques (65). Present study revealed that rBCG-DisA i.v immunization led to lower specific IgG than BCG (**Figures 5B, C**, **S3A**), which was inconsistent with our previous research by s.c route (27). While, anti-DisA antibody levels raised than that of BCG after *M. tuberculosis* infection (**Figure 6B**), which further confirmed strong immunogenicity of DisA as our previous work (27). Here, i.v rBCG-DisA led to the increasing of CD4 T cells in the spleen of C57BL/6J mice compared with BCG after *M. tuberculosis* infection (**Figure 6C**), as that of by s.c route in BALB/c mice (27). Unexpectedly, rBCG-DisA immunized mice showed a significant increase CD4 T cells in the lung after *M. tuberculosis* infection, which suggested that enhanced cellular immune response induced in the lung of mice. Besides, our recent work showed that elevated c-di-AMP regulated expressions of several immune-associated proteins, and increased immunogenicity of *Mycobacterium smegmatis* by s.c route in mice (30). Hence, immune responses was induced by rBCG-DisA i.v immunization may be the results of the dual functions of overexpressed DisA and c-di-AMP regulated proteins.

Above, we had showed that rBCG-DisA i.v immunization could induce enhanced trained immunity as well as adaptive immunity in mice, especially accumulated T cells in the lung (**Figure 6D**). We found that rBCG-DisA i.v immunization could retain the body weight compared with BCG in mice (**Figure 5A**), but did not observe adverse reactions during the experiment, and pathological damage by H&E staining (**Figure 7A**). As well, H37Ra can be used as a surrogate for studying *M. tuberculosis* virulence in biosafety level 2 (BSL2) facilities (66). Previous, we showed that *M. tuberculosis* H37Ra and H37Rv were capable to induce comparable levels of humoral and cellular immunity by i.v route (67). The protection efficiency data showed that rBCG-DisA i.v vaccination promoted the clearance of *M. tuberculosis* H37Ra to undetectable in the lung and spleen of mice as BCG (**Figure 7B**). It was noted that rBCG-DisA rather than BCG was capable of long-term persistence in the lung after *M. tuberculosis* infection (**Figure 7C**). The longer resident of rBCG-DisA in the lung of mice may explain elevated T cell responses (**Figure 6D**, **7D**) and enhanced epigenetic modification, especially immune memory related H3K4me1 (**Figure 3A**). While, BMDMs from both rBCG-DisA and BCG immunized mice showed similar proinflammatory cytokine responses and protection against *M. tuberculosis* H37Ra infection (**Figures S5A–D**). It has been elucidated that BMDMs from BCG vaccinated mice could inhibit intracellular survival of virulent H37Rv (33). We speculate that the virulence and MOI of *M. tuberculosis* strain caused differences in results. Together, these results showed that rBCG-DisA may provide longer protective after *M. tuberculosis* infection, which was attributed to enhanced T cell immunity as well as trained immunity (3, 39).

It was reported that the protection efficacy of BCG vaccination at birth rarely persists beyond 15-20 years in TB epidemic regions, but highly variable in adults (68, 69). Currently, several new mycobacterial vaccine designs that aimed to improve the efficacy over BCG have been evaluated in animal models and some in humans, such as boosting BCG with homologous BCG or heterogeneous vaccines such as subunit vaccine of H4:IC31 (70). In view of the waned protective efficiency after one year of BCG vaccination (10, 71), we had a try to boost BCG-primed mice of 58 weeks with rBCG-DisA by s.c route (**Figure 8A**). Surprisingly, we found that rBCG-DisA boosting could help mice resist weight loss and significantly prolonged survival of mice after *M. tuberculosis* i.n challenge (**Figures 8B, C**). Regrettably, our prime-boost experiment was lack of a control group of un-primed mice. Epidemiological data suggest that BCG vaccination is safe and decreases the infections of all causes in the elderly, especially respiratory tract infections (7, 72). Hence, our results suggested a promising potential of rBCG-DisA in prime-boost strategy, especially in adults.

In present study, enhanced immune responses including trained immunity and adaptive immune response induced by rBCG-DisA i.v immunization were investigated. rBCG-DisA induced innate immunity including potent proinflammatory cytokine responses, epigenetic modification, altered lncRNA and metabolic profile. These results suggested that rBCG-DisA induced enhanced trained immunity, which may be stored within BM progenitor cells against infection over long periods. Additionally, vaccination to enhance trained immunity is a promising strategy which will be applied to adults or immunocompromised individuals due to defective adaptive immunity (52, 52, 54).

## Data availability statement

The raw data supporting the conclusions of this article will be made available by the authors, without undue reservation. The metabolome sequencing data presented in the study are deposited in the MetaboLights repository (URL https://www.ebi.ac.uk/metabolights/MTBLS5496), accession number MTBLS5496.

## Ethics statement

The animal study was reviewed and approved by The Institutional Ethics Committee of Second Affiliated Hospital of Air Force Medical University, using the recommendations from the Guide for the Care and Use of Laboratory Animals of the Institute (approval no. TDLL-20190213).

## Author contributions

HN, JK, YL, XL, RR, YZ, YX, LB, YK, XG, and MX performed experiments and analyzed data. JZ, SZ, LZ, and YM contributed cells, reagents and expertise. HN, YL, and YB wrote the manuscript. YB and FZ conceived and designed experiments. YB supervised this work. All authors have read and agreed with the data.

## Funding

This study was funded by National Natural Science Foundation (No. 81971560, 81371774, 81671638), National Major Special Projects of 13th Five-year Plan (No. 2018ZX10302302002004), Provincial Natural Science Foundation of Shaanxi Province (No. 2022ZDLSF01-07, 2019ZDLSF02-04), and Ningxia Natural Science Foundation (No. 2021AAC03124).

## Conflict of interest

The authors declare that the research was conducted in the absence of any commercial or financial relationships that could be construed as a potential conflict of interest.

The reviewer GD declared a shared affiliation, with no collaboration, with some of the authors, LZ, YK, XG, to the handling editor at the time of the review.

## Publisher’s note

All claims expressed in this article are solely those of the authors and do not necessarily represent those of their affiliated organizations, or those of the publisher, the editors and the reviewers. Any product that may be evaluated in this article, or claim that may be made by its manufacturer, is not guaranteed or endorsed by the publisher.
