# Supplementary material for: Cyclic di-AMP as endogenous adjuvant enhanced BCG-induced trained immunity and protection against Mycobacterium tuberculosis in mice
Source: Front Immunol. 2022 Aug 23;13:943667. doi: 10.3389/fimmu.2022.943667 (PMC9445367; doi:10.3389/fimmu.2022.943667)
Supplement: Supplementary file 1 [file DataSheet_1.docx]

**Table S1 Primers used in this study**

| **Sequence** | **Description** |
| --- | --- |
| **quantitative RT-PCR** | |
| acaactttggcattgtggaa | qRT-PCR primer of Mus *gapdh*, forward |
| gatgcagggatgatgttctg | qRT-PCR primer of Mus *gapdh*, reverse |
| gctctgagacaatgaacgctac | qRT-PCR primer of Mus *ifn-γ*, forward |
| tgcaggattttcatgtcacc | qRT-PCR primer of Mus *ifn-γ*, reverse |
| cttgtgctccttgtcaacag | qRT-PCR primer of Mus *il-2*, forward |
| tccaagttcatcttctaggc | qRT-PCR primer of Mus *il-2*, reverse |
| tgctcttactgactggcat | qRT-PCR primer of Mus *il-10*, forward |
| ctggatcatttccgataaggc | qRT-PCR primer of Mus *il-10*, reverse |
| agctccaagaaaggacgaaca | qRT-PCR primer of Mus *ifn-β*, forward |
| gccctgtaggtgaggttgat | qRT-PCR primer of Mus *ifn-β*, reverse |
| gaaatgccaccttttgacagtg | qRT-PCR primer of Mus *il-1β*, forward |
| tggatgctctcatcaggacag | qRT-PCR primer of Mus *il-1β*, reverse |
| cggccttccctacttcacaa | qRT-PCR primer of Mus *il-6*, forward |
| actccagaagaccagaggaa | qRT-PCR primer of Mus *il-6*, reverse |
| cctacctgcttctcacccatacc | qRT-PCR primer of Mus *tnf-α*, forward |
| ttgatggcagagagaaggttga | qRT-PCR primer of Mus *tnf-α*, reverse |
| ggagttcgaggaaccctagtg | qRT-PCR primer of Mus *cxcl9*, forward |
| gggatttgtagtggatcgtgc | qRT-PCR primer of Mus *cxcl9*, reverse |
| ccaagtgctgccgtcattttc | qRT-PCR primer of Mus *cxcl10*, forward |
| ggctcgcagggatgatttcaa | qRT-PCR primer of Mus *cxcl10*, reverse |
| tgtaatttacccgagtaacggc | qRT-PCR primer of Mus *cxcl11*, forward |
| cacctttgtcgtttatgagcctt | qRT-PCR primer of Mus *cxcl11*, reverse |
| tcgagaccatttactgcaacag | qRT-PCR primer of Mus *cxcl15*, forward |
| cattgccggtggaaattcctt | qRT-PCR primer of Mus *cxcl15*, reverse |
| cagctgggctgtacaaaccttc | qRT-PCR primer of Mus *inos*, forward |
| cattggaagtgaagcgtttcg | qRT-PCR primer of Mus *inos*, reverse |
| aaggaagcttggcgttgtga | qRT-PCR primer of Mus *lincRNA-Cox2*, forward |
| gagaggtgaggagtcttatg | qRT-PCR primer of Mus *lincRNA-Cox2*, reverse |
| cttgttctgggagcatcat | qRT-PCR primer of Mus *lncRNA NEAT1* (short+long), forward |
| ctacaccttacgcaatcttct | qRT-PCR primer of Mus *lncRNA NEAT1* (short+long), reverse |
| tcctctacagccttacctacatc | qRT-PCR primer of Mus *lncRNA NEAT1*-2 (long), forward |
| agacaaccttcaaccaacaacc | qRT-PCR primer of Mus *lncRNA NEAT1*-2 (long), reverse |
| gagggaaggtcaagggtcaa | qRT-PCR primer of Mus *lncRNA NeST*, forward |
| gcgagatccccacatcctaa | qRT-PCR primer of Mus *lncRNA NeST*, reverse |
| ttcaaacggcttagcaccct | qRT-PCR primer of Mus *lncRNA NRON*, forward |
| tgtcctgctgggaccagata | qRT-PCR primer of Mus *lncRNA NRON*, reverse |
| **PCR verification for bacteria** | |
| atgacagacgtgagccga | Primer of *Rv1886c* (*ag85B*), forward |
| tcagccggcgcctaacga | Primer of *Rv1886c* (*ag85B*), reverse |
| atggcagagatgaagacc | Primer of *Rv3874-Rv3875* (*cfp10-esat-6*) operon, forward |
| ctatgcgaacatcccagt | Primer of *Rv3874-Rv3875* (*cfp10-esat-6*) operon, reverse |

**
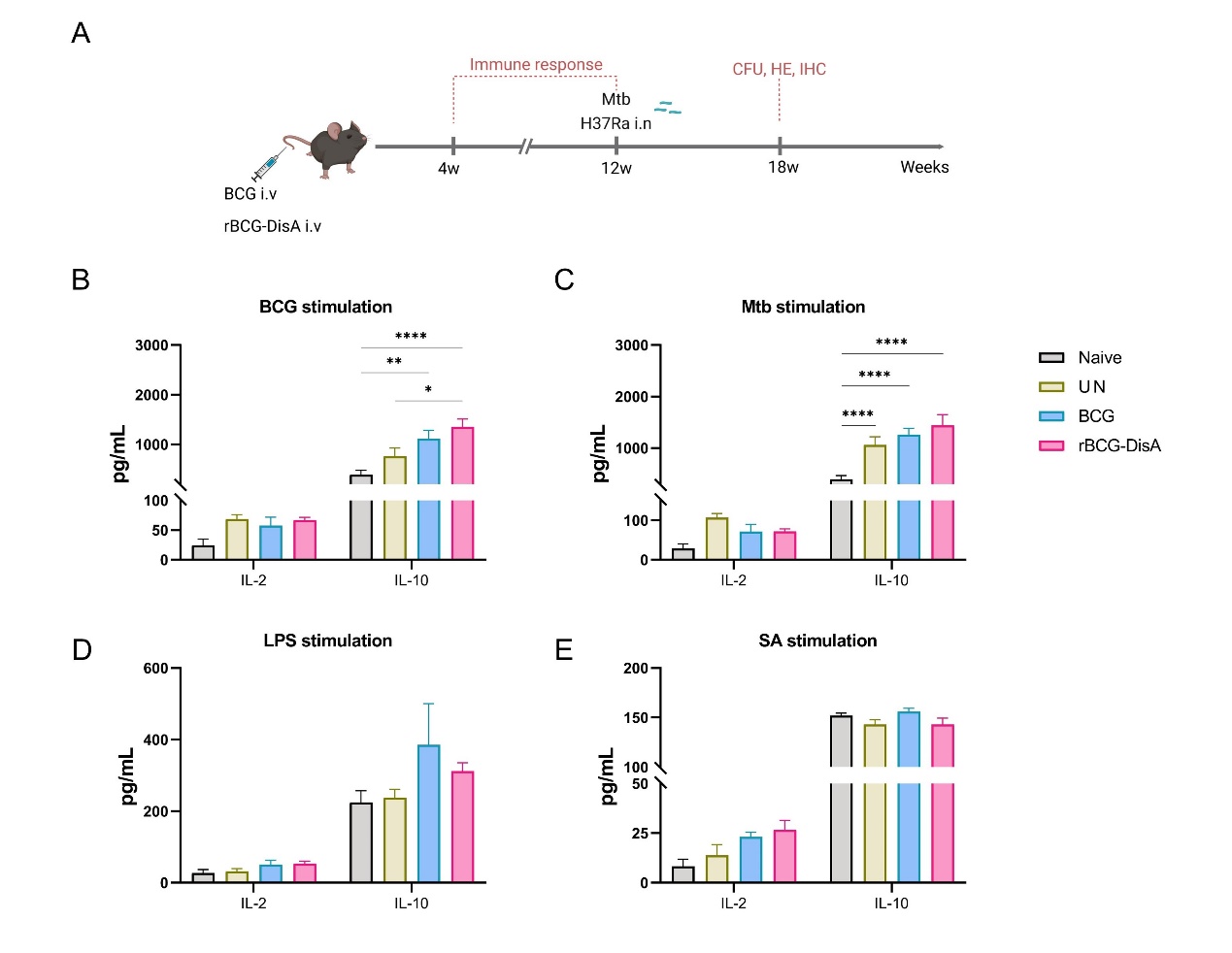
**

**Figure S1 Inflammatory cytokines production by splenocytes of vaccinated mice after *M. tuberculosis* i.n infection. (A)** Schematic representation of the timeline of vaccination and challenge strategies (created with BioRender). Mice were vaccinated by i.v BCG and rBGC-DisA for 12 weeks. Then, mice were infected with *M. tuberculosis* by i.n route, infected mice without immunization (UN) were used as control. At 6 weeks post infection, immune responses were detected. (**B-E**) At 6 weeks post infection, splenocytes were isolated and re-stimulated with BCG protein extracts (BCG) (25 μg/mL) **(B)**, *M. tuberculosis* (Mtb) (25μg/mL) **(C)**, *E. coli* O111:B4 LPS (LPS) (100ng/mL) **(D)**, and *S. aureus* (SA) (25μg/mL) **(E)** for 72h *in vitro*. Cytokines production of IL-2 and IL-10 in supernatants were determined by ELISA (*n*=4). (^*^*P* < 0.05, ^**^*P* < 0.01, ^***^*P* < 0.001, ^****^*P* < 0.0001.)


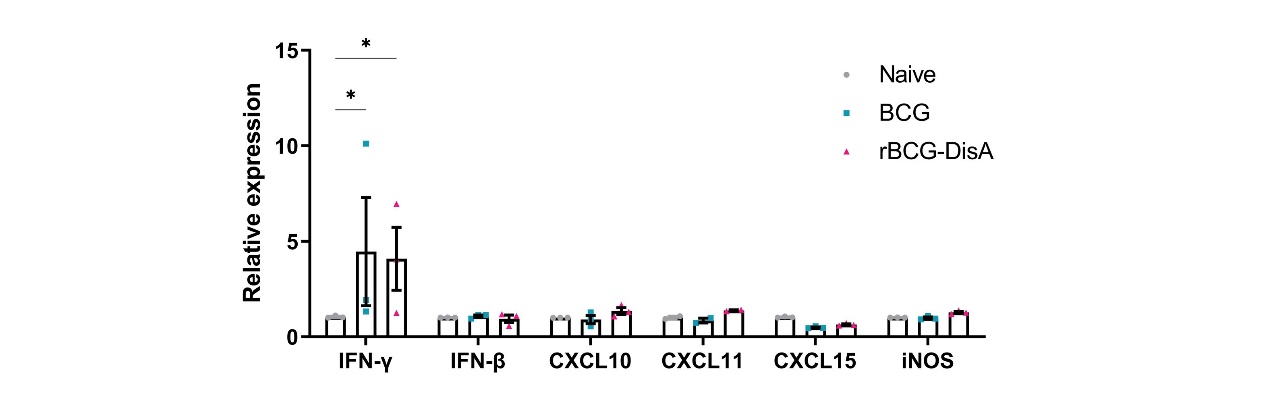


**Figure S2 Expression of inflammation-related genes in BMs of vaccinated mice.** At 8 weeks after BCG and rBCG-DisA i.v immunization, bone marrow (BM) cells were isolated and the expression of indicated inflammation-related genes were detected by quantitative RT-PCR (*n*=3). (^*^*P* < 0.05.)


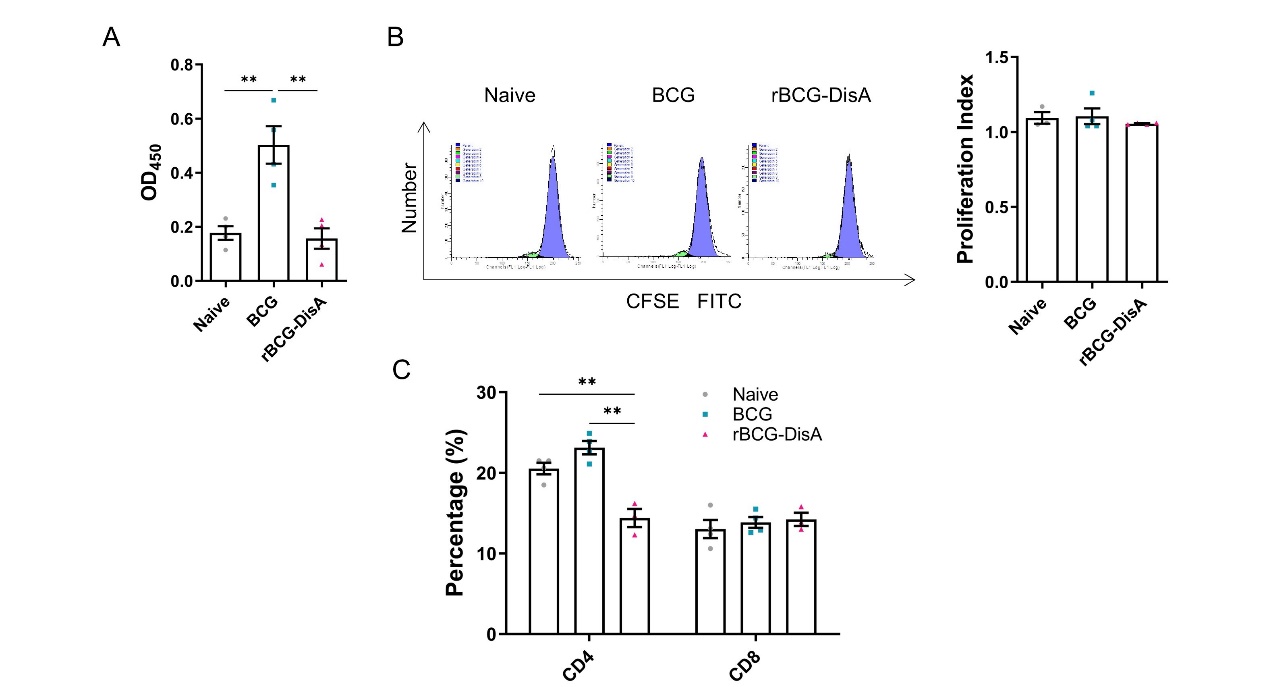


**Figure S3 Humoral and cellular immune responses in immunized mice.** **(A)** After 12 weeks of i.v immunization, BCG-specific IgG levels in mice sera (1:200) were assayed by ELISA (*n*=4). **(B)** After 4 weeks of i.v immunization, splenocytes were labeled with CFSE and cell proliferation was measured by flow cytometry after BCG antigens stimulation (left). Proliferation index was quantitatively analysed by Modfit software (*n*=4) (right). **(C)** After 4 weeks of i.v immunization, proportions of CD4 and CD8 T cells in splenocytes were measured by flow cytometry (*n*=4). (^**^*P* < 0.01.)


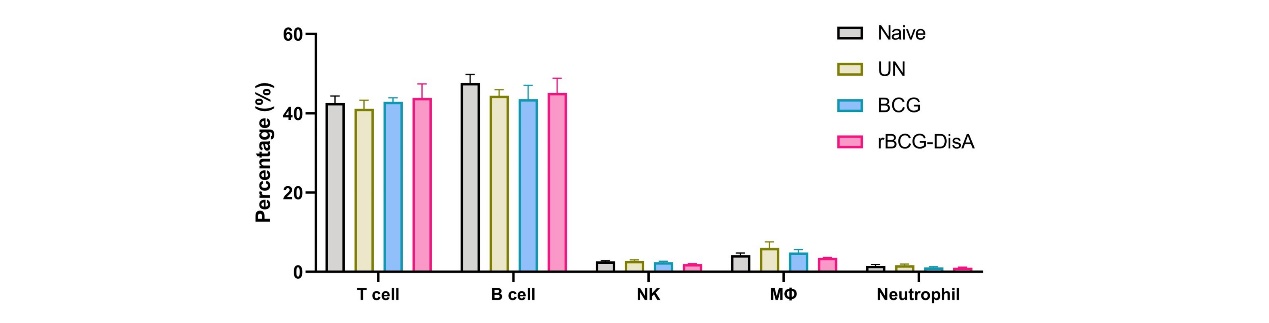


**Figure S4 Immune cell subsets cells of immunized mice after *M. tuberculosis* infection.** Mice were vaccinated by BCG and rBGC-DisA for 12 weeks. Then, mice were infected with *M. tuberculosis* by i.n route, infected mice without immunization (UN) were used as control. At 6 weeks post infection, proportions of immune cell subsets cells as indicated in splenocytes were assayed by flow cytometry (*n*=4).


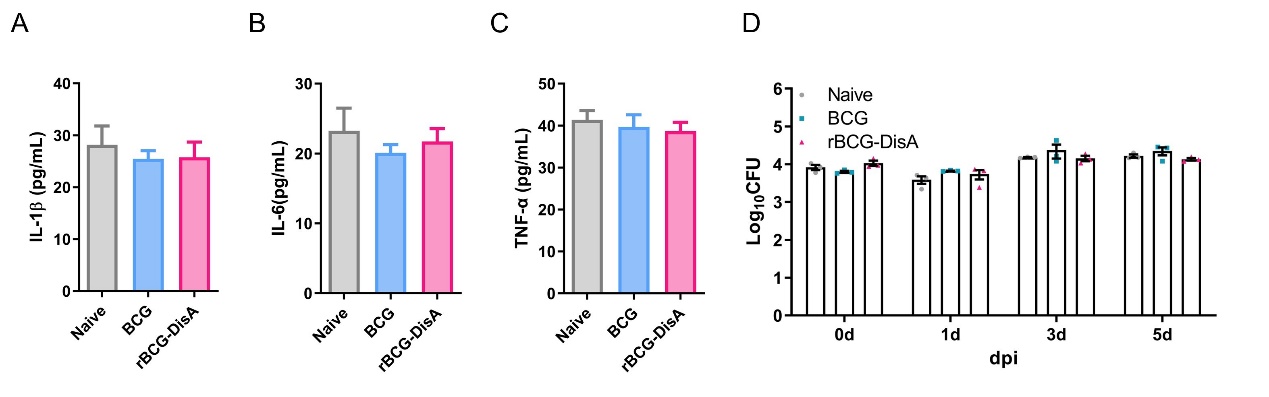


**Figure S5 Proinflammatory cytokines production by BMDMs after *M. tuberculosis* infection and intracellular survival of bacilli.** After 4-week of i.v vaccination, BM were isolated from BCG and rBCG-DisA vaccinated mice and induced differentiation into macrophages (BMDMs). Normal mice (Naive) were used as control. BMDMs were infected with a *M. tuberculosis* attenuated strain H37Ra at MOI=1. **(A-C)** After 24h infection, supernatants were collected to determine the production of IL-1β **(A)**, IL-6 **(B)** and TNF-α **(C)** by ELISA. **(D)** The intracelluar survival capacity of *M. tuberculosis* was assessed at 0-, 1-, 3- and 5-day post infection (*n*=3).
